# Genetic diversity of the merozoite surface protein-3 gene in *Plasmodium falciparum* populations in Thailand

**DOI:** 10.1186/s12936-016-1566-1

**Published:** 2016-10-21

**Authors:** Sittiporn Pattaradilokrat, Vorthon Sawaswong, Phumin Simpalipan, Morakot Kaewthamasorn, Napaporn Siripoon, Pongchai Harnyuttanakorn

**Affiliations:** 1 Department of Biology, Faculty of Science, Chulalongkorn University, Bangkok, 10330 Thailand; 2 Department of Pathology, Faculty of Veterinary Science, Chulalongkorn University, Bangkok, 10330 Thailand; 3 College of Public Health Sciences, Chulalongkorn University, Bangkok, 10330 Thailand

**Keywords:** DNA sequencing, Genetic diversity, Vaccine, Merozoite surface protein, Southeast Asia

## Abstract

**Background:**

An effective malaria vaccine is an urgently needed tool to fight against human malaria, the most deadly parasitic disease of humans. One promising candidate is the merozoite surface protein-3 (MSP-3) of *Plasmodium falciparum*. This antigenic protein, encoded by the merozoite surface protein (*msp*-*3*) gene, is polymorphic and classified according to size into the two allelic types of K1 and 3D7. A recent study revealed that both the K1 and 3D7 alleles co-circulated within *P. falciparum* populations in Thailand, but the extent of the sequence diversity and variation within each allelic type remains largely unknown.

**Methods:**

The *msp*-*3* gene was sequenced from 59 *P. falciparum* samples collected from five endemic areas (Mae Hong Son, Kanchanaburi, Ranong, Trat and Ubon Ratchathani) in Thailand and analysed for nucleotide sequence diversity, haplotype diversity and deduced amino acid sequence diversity. The gene was also subject to population genetic analysis (*F*_*st*_) and neutrality tests (Tajima’s *D*, Fu and Li D* and Fu and Li’ F* tests) to determine any signature of selection.

**Results:**

The sequence analyses revealed eight unique DNA haplotypes and seven amino acid sequence variants, with a haplotype and nucleotide diversity of 0.828 and 0.049, respectively. Neutrality tests indicated that the polymorphism detected in the alanine heptad repeat region of MSP-3 was maintained by positive diversifying selection, suggesting its role as a potential target of protective immune responses and supporting its role as a vaccine candidate. Comparison of MSP-3 variants among parasite populations in Thailand, India and Nigeria also inferred a close genetic relationship between *P. falciparum* populations in Asia.

**Conclusion:**

This study revealed the extent of the *msp*-3 gene diversity in *P. falciparum* in Thailand, providing the fundamental basis for the better design of future blood stage malaria vaccines against *P. falciparum*.

**Electronic supplementary material:**

The online version of this article (doi:10.1186/s12936-016-1566-1) contains supplementary material, which is available to authorized users.

## Background

Vaccines against human-infecting *Plasmodium* species are considered an indispensible tool for malaria control and eradication. To date, RTS/S is the most advanced malaria vaccine against the human malaria parasite *Plasmodium falciparum,* the most deadly of the human malaria parasites. The RTS/S vaccine is made from the circumsporozoite protein (CSP), the essential antigen of the sporozoite, and induces immunity against the pre-erythrocytic stage of the malaria parasite. Although the vaccine has completed the pivotal Phase III clinical testing, and subsequently received a positive scientific recommendation from European Medical Agency for immunization of children against malaria in 2015, the efficacy of vaccine against clinical malaria was less than 50 % in children and infants [1].

An alternative approach for malaria intervention is to develop vaccines against the pathogenic erythrocytic stages, aiming to reduce their ability to induce morbidity and mortality. The erythrocyte invasion is a critical point in the life cycle of malaria, and at this point the merozoites can be targeted by antibodies and subsequently eliminated by white blood cells. This type of vaccine generally incorporates antigenic proteins that are highly expressed on, or associated with, the surface of merozoites, such as the merozoite surface proteins (MSPs). All current erythrocytic stage vaccines are now being tested in clinical Phase I and II trails, yet in many cases the vaccines have had low, or no, impact against clinical malaria [2]. One of the key difficulties for malaria vaccine development is the structural diversity of the malaria antigens that is caused by the extensive genetic polymorphism of the antigen-encoding genes. This is attributed to high mutation and genetic recombination rates, features commonly observed in areas of high malaria endemicity. The analyses of *P. falciparum* diversity in natural infections by high-throughput sequencing, microarrays and genome-wide microsatellite markers revealed that the *P. falciparum* genome is highly diverse [3–5]. A complete understanding of the malaria parasite’s biology and the extent of genetic diversity in natural parasite populations will be necessary for improving vaccine design and efficacy.

This study will focus on MSP-3, a promising candidate antigen for the development of a blood-stage malaria vaccine. The MSP-3 protein has a crucial role not only in binding to the host red blood cell, but also in protecting the parasite against haem that is released during parasite egression [6]. The MSP-3 protein is synthesized as 62 kDa precursor at the schizont stage and is secreted into the parasitophorous vacuole (PV), where it undergoes cleavage at its N-terminal sequence to form the mature processed form, with a final size of 44–48 kDa [7, 8]. Despite a lack of a transmembrane domain or Glycosylphosphatidylinisotol (GPI)-anchor containing sequence, the protein is presumed to attach to the surface membrane as a result of protein–protein interactions. The N-terminal region of MSP-3 is defined by three blocks of four alanine heptad repeats (AHRs), with the conserved motif ‘AXXAXXX’, which are separated by short stretches of non-repetitive sequences [9]. The alanine residues in the AHRs are thought to stabilize the α-helical secondary structure [10]. The C-terminal region of MSP-3 comprises a glutamic acid-rich region and a putative leucine zipper sequence, which are important for trafficking of MSP-3 and its binding partners to the PV [11].

Importantly, evidence that the *P. falciparum* MSP-3 protein is a target of natural acquired immunity is accumulating. First, hyperimmune sera that inhibited *P. falciparum* growth in vitro in the presence of blood monocytes in an antibody-dependent cellular assay were shown to contain antibodies targeting MSP-3 [12, 13]. Subsequently, MSP-3-specific human antibodies were shown to suppress *P. falciparum* growth in immunocompromised mice infected with *P. falciparum* [14]. Immunization with the full-length sequence of *P. falciparum* MSP-3 also induced a protective immune response and protected *Aotus* monkeys from subsequent challenge infection with *P. falciparum* expressing a homologous MSP-3 sequence [15]. Antibodies targeting MSP-3 are also associated with long-term protection against clinical malaria [16]. Vaccines have been now designed using either MSP-3 alone or in combination with other blood-stage antigens, such as the glutamate rich protein (GLURP) or MSP-1. For example, the MSP-3/LPS vaccine is a long synthetic peptide, consisting of the C-terminal portion (corresponding to amino acid positions 154–249) of *P. falciparum* strain FC27, which has been tested in Phase I clinical trials [17, 18]. The vaccine was shown to be safe, immunogenic and capable of inducing cytophilic antibodies that could inhibit *P. falciparum* erythrocytic growth in a monocyte-dependent manner, under both in vitro and in vivo conditions [19–21]. The GMZ2 vaccine, which consists of a hybrid protein between the C-terminal MSP-3 fragment (corresponding to amino acid positions 212–318 of *P. falciparum* 3D7 strain) and the N-terminal region of GLURP, has recently been developed and evaluated during Phase I trials [22–24]. Like the MSP-3 LPS vaccine, GMZ2 was immunogenic and well tolerated, and elicited high titres of functional antibodies with the capacity to control *P. falciparum* multiplication [25]. Additionally, several studies showed that the N-terminal sequences were immunologically important and could potentially be considered as a sub-unit of a malaria vaccine [26, 27]. Altogether, these studies support that the N and C-terminal regions of MSP-3 are targets of protective responses and so are considered promising malaria vaccine candidates.

The *P. falciparum* MSP-3 protein is encoded by a single locus (PF10_0345; *msp*-*3*) that spans 1.1 kb on chromosome 10 [28]. Orthologs of the *P. falciparum msp*-*3* gene were identified in malaria parasites of African apes *Plasmodium reichenowi* and *Plasmodium gaboni* [29, 30], not in the human malaria parasite *Plasmodium vivax* and Asian primate malarias [31]. Comparison of *msp*-*3* sequences from laboratory strains of *P. falciparum* revealed a dimorphic pattern, with allelic sequences falling into the two major classes of 3D7 and K1 (named after the *P. falciparum* strains) [32]. Variation between the two classes of *msp*-*3* is largely due to insertion/deletion (indel) and nucleotide substitutions within and flanking the AHR domains, which define the N-terminal domain. Conversely, the C-terminal domain is almost entirely conserved [9, 32]. The two allelic types (3D7 and K1) of the *msp*-*3* gene could be genotyped by agarose gel electrophoresis based size estimation of the polymerase chain reaction (PCR) amplification of the *msp*-*3* gene. Allelic diversity of the *P. falciparum msp*-*3* gene was also studied in natural parasite populations, providing a snapshot of genetic diversity in diverse geographical populations [33–35]. Alternatively, the genomes of *P. falciparum* natural isolates have been sequenced, providing the systematic view of polymorphic sites at *msp*-*3* locus, but the variation within allelic type of *msp*-*3* has been rarely analysed [3, 4, 36]. Although one study described the sequence variation of the *msp*-*3* gene in wild isolates of *P. falciparum* in Thailand and Nigeria [37], the *msp*-3 sequence data from Thailand was obtained from a single population in Tak at the Thailand–Myanmar border, and, to date, there is no report of sequence variation with allelic type of *msp*-*3* in other regions of Thailand or other countries in Southeast Asia.

To address this question, the *msp*-*3* sequences from natural Thai isolates from five known malaria hotspots, near the borders between Thailand and three neighbouring countries (Cambodia, Myanmar, Laos) were examined. These data provide an overview of the *msp*-*3* variants and population structure of *P. falciparum* across regions of Thailand, and aid in identifying regions under positive selection. This should provide the basis for a better design of MSP-3-based malaria vaccines.

## Methods

### Origins of *Plasmodium falciparum*

A total of 59 natural isolates of *P. falciparum* used in the present study were the laboratory lines maintained at Malaria Laboratory, Department of Biology, Faculty of Science, Chulalongkorn University, Thailand. The parasites were originally isolated from patients in the five endemic areas of Mae Hong Son, Kanchanaburi and Ranong at the Thailand–Myanmar border, Ubon Ratchathani at the Thailand–Laos border, and Trat at the Thailand–Cambodia border between 2002 and 2010 [38]. The procedures of parasite collection and in vitro cultivation were performed as previously described [39]. Microscopic examinations of Giemsa-stained thin blood smears were performed to identify the *Plasmodium* species. The parasites were previously genotyped with microsatellites and shown to be genetically distinct clones [39], and with primers specific to the *msp*-*3* gene [28]. Those *P. falciparum* isolates shown to have a single allele of *msp*-*3* were chosen for this study.

### Genomic DNA preparation, nested PCR and DNA sequencing

Genomic DNA was prepared from infected human blood using a standard phenol/chloroform extraction method [38]. In brief, the blood stage parasites of *P. falciparum*, with 10–20 % parasitaemia were harvested and incubated with 0.05 % (w/v) saponin solution in phosphate buffered saline (PBS, pH 7.4). The parasites were lysed in buffer (40 mM Tris–HCl, 80 mM EDTA, 2 % (w/v) sodium dodecyl sulfate (pH 8.0), supplied with 2 mg/mL proteinase K) at 42 °C overnight. The lysate was mixed with Tris–HCl saturated phenol (pH 8.0) to precipitate proteins and, after phase separation by centrifugation, the aqueous phase was harvested. The lysate was then mixed with phenol/chloroform/isoamyl alcohol (25:24:1 (v/v/v), pH 8.0), phase separated as above and the aqueous phase harvested. The DNA was precipitated by addition of a 0.1× volume of 0.3 M sodium acetate (pH 5.2) and a 1× volume of absolute ethanol and centrifugation. The genomic DNA pellets were washed with 70 % (v/v) ethanol and dissolved in TE buffer (10 mM Tris–HCl, 0.1 mM Na_2_EDTA, pH 8.0) and stored at −20 °C.

The *msp*-*3* gene was amplified by two-stage nested PCR as follows. The first PCR reaction was performed in a total volume of 50 µl, containing 200-300 ng of DNA template, 2 mM of MgCl_2_, 200 µM of dNTPs, 0.5 µM of each primer (M3F/O and M3R/O) and 2 units (U) of FastStart *Taq* DNA polymerase enzyme in 1X *Taq* PCR buffer (Roche Diagnostics, Germany). Thermal cycling was performed with an optimized profile of 95 °C for 5 min, followed by 40 cycles of 95 °C for 40 s, 56 °C for 40 s and 68 °C for 80 s, and then a final 68 °C for 10 min. The PCR products were diluted 100- or 500-fold in distilled water and used as the DNA template for the second PCR reaction. The second PCR reaction was performed in a total volume of 150 µl of the same composition as above except for the template and the M3R/I primer was used in place of M3R/0. Thermal cycling was performed with an optimized profile of 95 °C for 5 min, followed by 30 cycles of 95 °C for 45 s, 57 °C for 45 s and 68 °C for 80 s, and then a final 68 °C for 7 min. The primer sequences (M3F/O 5′-ATGAAAAGTTTTAT AAATATTACTCTTTC-3′, M3R/O 5′-CATGTTATGAATATAAATTATGTTCA-3 and M3R/I 5′-AATGATTTTTAAAATATTTGGATAATTC-3′) correspond to nucleotide positions 1,404,192–1,404,220 (the start codon at position 1,404,192–1,404,194), 1,405,293–1,405,268 (the stop codon at position 1,405,254–1,405,256) and 1,405,254–1,405,227, of the chromosome 10 of *P. falciparum* strain 3D7 (NCBI accession number: AE014185.2) [30]. The PCR products were analysed by agarose gel electrophoresis. Sequencing reactions were performed using BigDye Terminator v1.1 kit (Applied Biosystems) with an ABI3730 DNA analyser. DNA sequences were manually edited using BioEdit 7.0.0 software and aligned using the multiple sequence alignment program Clustal Omega [40].

### Phylogenetic analysis

Alignment of nucleotide sequences of *msp*-*3* gene was performed using BioEdit 7.0.0 software. Segments for which a reliable alignment could not be inferred were eliminated. Phylogenetic tree searching was implemented using the maximum likelihood (ML) method in MEGA V6.0. The Bayesian information criterion (BIC), as implemented in MEGA V6.0, was used to identify the best-fit model of sequence evolution for the trees estimated using ML. The evolutionary history was inferred using the ML method based on the general time reversible with the Hasegawa-Kishino-Yano with gamma distribution shape parameter (HKY + G) model. The robustness of ML trees was evaluated by bootstrap analysis of 1000 replicates. The *msp*-*3* gene sequence of the chimpanzee malaria parasite *Plasmodium reichenowi* (NCBI accession number: HG810771 [41]) was used as an out group.

### Sequence analysis and statistical analysis

Several measures of genetic polymorphism and neutrality tests were calculated using the MEGA software [42], including the number of polymorphic sites (S), nucleotide diversity (π) as the average number of nucleotide substitutions per site between any two sequences, the number of haplotypes (H), and haplotype diversity (Hd). The nucleotide diversity was also plotted using a sliding window with a window length of 60 bases and step size of 3 bp, in the DnaSP 5.0 software [43] to identify the region(s) of *msp*-*3* that accumulate polymorphisms. The mean number of synonymous mutations per synonymous site (*d*_*S*_) and non-synonymous substitutions per non-synonymous site (*d*_*N*_) within each isolate were calculated using the Nei and Gojobori method [44], with the Jukes and Cantor correction, and the statistical differences between *d*_*N*_ and *d*_*S*_ were tested with the Z test of selection (*P* < 0.05). A *d*_*N*_ */d*_*S*_ ratio of greater than 1 at the 95 % confidence interval was taken as evidence of positive diversifying selection. In addition, three population genetic tests of neutrality (Tajima’s *D*, Fu and Li’D* and Fu and Li’F* tests) were applied to the *msp*-*3* sequences to determine whether polymorphism takes place at higher or lower frequencies than expected under a neutral model [45, 46]. In addition, sliding window plots, with a window length of 60 bases and a step size of 3 bp, were generated for the analysis of the three neutrality tests above to identify regions of *msp*-*3* where a significant departure from neutrality was observed (*P* < 0.05). Differences in the distribution patterns (ratios) of MSP-3 variants between different geographic populations of *P. falciparum* were tested using Wright’s fixation index (*F*_*st*_) in the Arlequin suite version 3.5 software [47], accepting significance at the *P* < 0.05 level.

## Results

### Nucleotide sequence analysis of the *msp*-*3* gene of *Plasmodium falciparum* in Thailand

Fragments of the *P. falciparum msp*-3 gene were successfully amplified from 59 human blood samples collected from five endemic areas. Thirty-seven samples were obtained from three localities at the Thailand–Myanmar border, as 12, 13 and 12 isolates from Mae Hong Son, Kanchanaburi and Ranong, respectively. In addition, 12 and ten samples were obtained from Ubon Ratchatani and Trat, which were situated at the Thailand–Laos border and the Thailand–Cambodia border, respectively. The derived PCR products were analysed by standard agarose gel electrophoresis, as described in Methods, revealing that 17 and 42 samples had the 3D7 and K1 allelic types of *msp*-*3* (Additional file 1), respectively, giving frequencies of the 3D7 and K1 allelic types of 28.8 and 71.2 %, respectively.

**Fig. 1:**
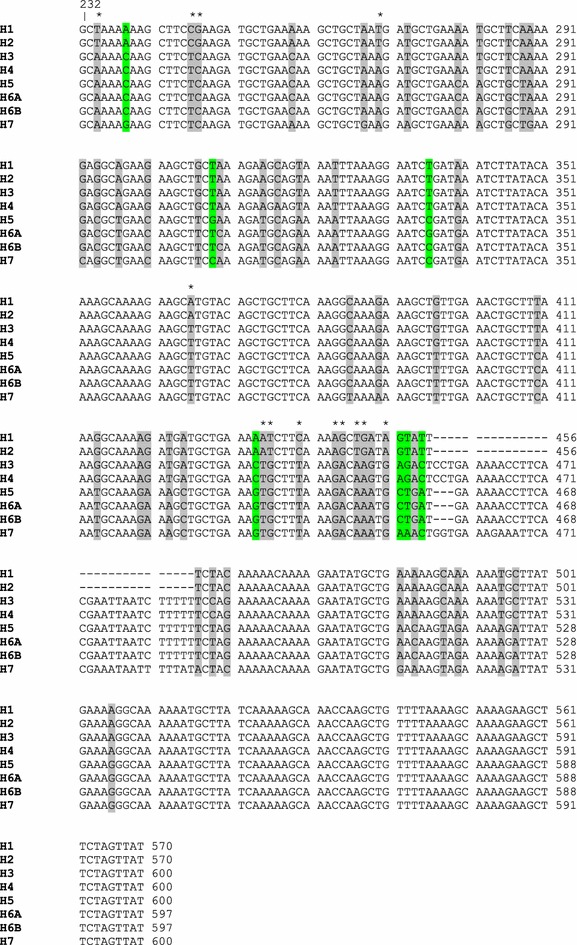
Partial nucleotide sequences of the *msp*-*3* gene of *Plasmodium falciparum* populations in Thailand. Only the nucleotide sequences encoding the AHRs of MSP-3, corresponding to nucleotide positions 232–570 after *P. falciparum* strain 3D7, are shown. H1 and H2 are derived from the 3D7 allele of *msp*-*3*, while the remaining 6 haplotypes (H3–5, H6A, H6B and H7) belong to the K1 allelic type. Two haplotypes, H6A and H6B, encode identical amino acid sequences (see Fig. 4).* Grey* and* green* labels indicate dimorphic and trimorphic nucleotides, respectively. *Asterisks* indicate dimorphic SNP defining the 3D7 and K1 alleles

**Fig. 2:**
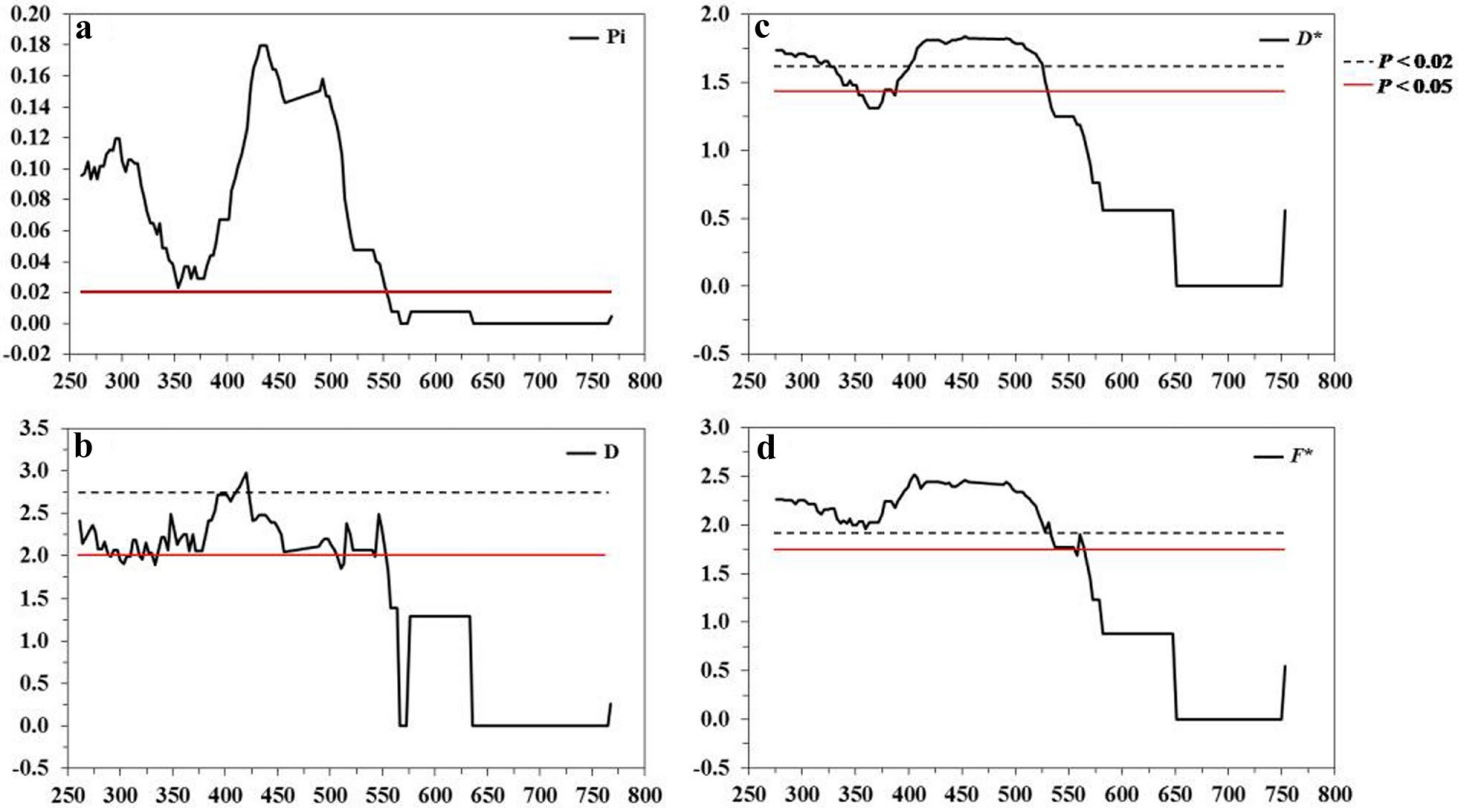
Sliding window plots of the average pair-wise nucleotide diversity (π), Tajima’s *D* values, Fu and Li’s D* values and Fu and Li’s F* values of the *msp*-*3* gene of *Plasmodium falciparum* in Thailand. **a** The nucleotide diversity was plotted by a sliding window with a window size of 60 bp. and a step size of 3 bp. The maximum diversity (**π** > 0.02) was detected between nucleotide positions 252–552 bp. Sliding window plots of **b** Tajima’s *D* values, **c** Fu and Li’s D* values and **d** Fu and Li’s F* values were performed with a window size of 60 bp. and a step size of 3 bp. Elevated *D* and F* values were detected between nucleotide positions 252–552 bp. and 252–561 bp., respectively. Significant *D* values were located between nucleotide positions 252–528, excluding the nucleotide positions 351–375 bp.* Red* and* dash lines* indicate *P* values of <0.05 and <0.02

A total of 59 partial sequences of *msp*-*3*, corresponding to nucleotide positions 232–783 bp (numbered from the *P. falciparum* reference strain 3D7 sequence) were generated and used in subsequent analyses. The sequence data contain the three blocks of the AHR region and the glutamic-rich region. A total of 60 polymorphic nucleotide sites (excluding gaps) and two indels were observed among the 59 sequences, with an average pairwise nucleotide diversity (π) of 0.049. Of these polymorphic sites, 53 were dimorphic and seven were trimorphic (Fig. 1). A sliding method plot with a window length of 60 bp and a step size of 3 bp revealed a π diversity range from 0.0001–0.1797. Significant values, which indicate the region of *msp*-*3* accumulating polymorphism, were observed between nucleotide positions 252–552 (Fig. 2a).

**Fig. 3:**
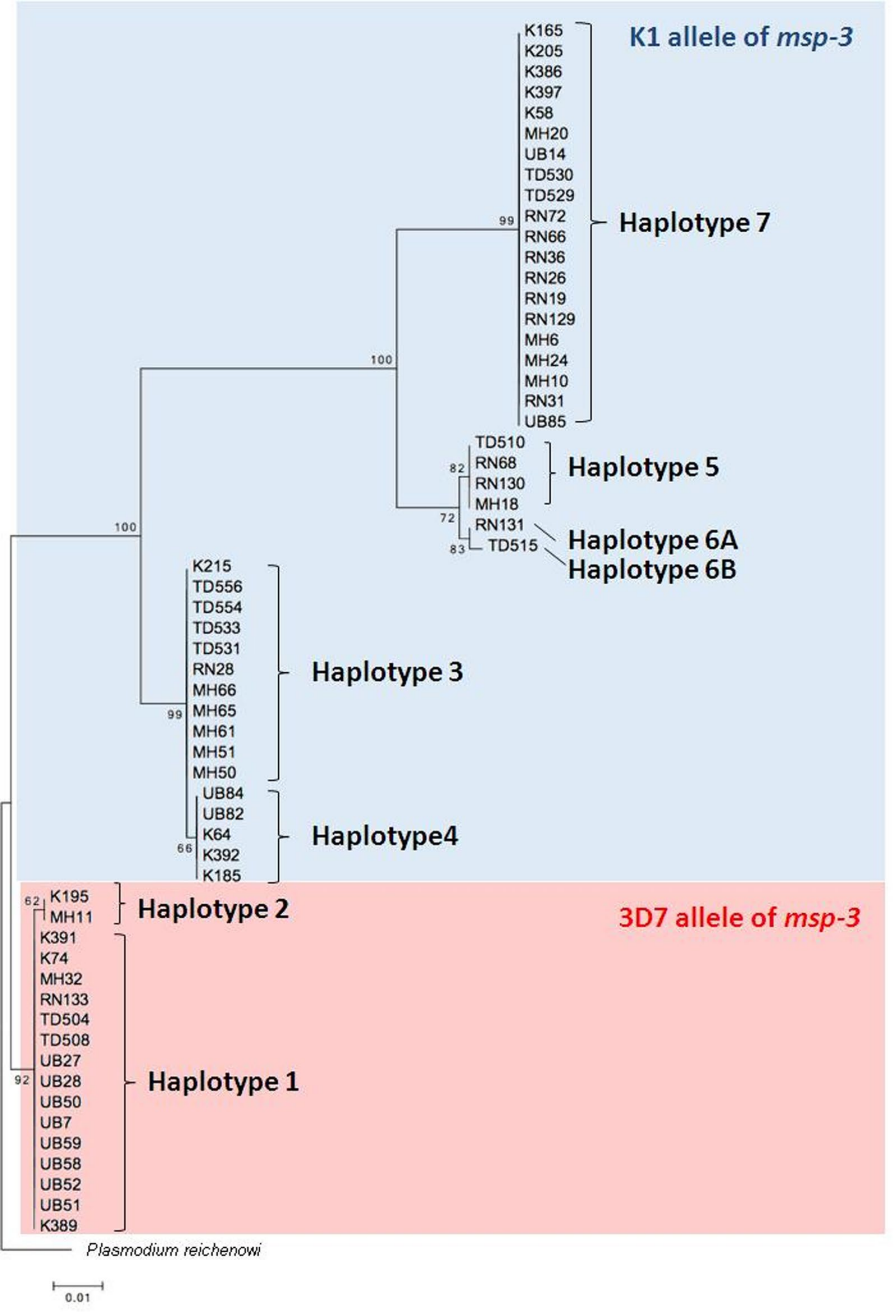
Maximum likelihood tree of Thai *Plasmodium falciparum* populations based on *msp*-*3* gene analysis. The tree was constructed by the MEGA program using *msp*-*3* nucleotide sequences (nucleotide positions 232–783 after *P. falciparum* 3D7 strain). The sequences are identified with their names of parasite isolates. The* letters* indicate the origin of isolates: MH, Mae Hong Son; K, Kanchanaburi; RN, Ranong; TD, Trat; UB, Ubon Ratchatani. The tree was rooted using the sequence of the *P. reichenowi msp*-*3* gene as an out group [41]. Bootstrap values of >50 % are shown. The *scale bar* indicates nucleotide substitutions per site

Nucleotide sequence alignment also revealed 8 distinguishable haplotypes (H1–5, H6A, H6B and H7; Fig. 1) with an estimated haplotype diversity (Hd) of 0.828. The ML-based phylogenetic analysis of these eight haplotypes is illustrated in Fig. 3. The 17 sequences with the 3D7 allele were represented by two haplotypes (H1 and H2), while the 42 sequences with the K1 allele were composed of six haplotypes. The H1 and H7 haplotypes had the nucleotide sequences of the reference strains 3D7 and K1, and were the most abundant haplotypes of *msp*-*3* in Thailand, with an overall frequency of 25 and 34 %, respectively, (Additional file 1).

Dimorphism of the 3D7 and K1 alleles of *msp*-*3* was attributed to variation in size of the AHR region, being 339 bp in the 3D7 allele and varying at 366 or 369 bp in the K1 allele. A 30-bp insertion fragment, corresponding to nucleotide positions 457–486 of the K1 strain, appeared in H3, H4 and H7, whereas a slightly shorter 27-bp insertion fragment, corresponding to nucleotide positions 457–483, was found in H5, H6A and H6B (Fig. 1). In addition to the above indels, 13 single nucleotide polymorphism (SNP) loci that were dimorphic but conserved within each allelic type were identified. Of these sites, four codons [234 (GCT/GCA), 246 (TCC/TCT), 366 (GCA/GCT) and 444 (AAA/AAG)] resulted in synonymous mutations at positions 76 (A), 82 (S), 122 (A) and 148 (K), while eight codons [247 (GAA/CAA), 270 (AAT/AAA/GAA), 435 (AAA/ACT/AGT), 436 (TCT/GCT), 440 (TCA/TTA), 445 and 447 (GCT/ACA), 448 (GAT/AGT/AAT) and 451 (AGT/GAG/GCT/GAA)] caused non-synonymous amino acid substitutions at positions 83 (E/Q), 90 (N/K/E), 145 (K/T/S), 146 (S/A), 147 (S/L), 149 (A/T), 150 (D/S/N), and 151 (S/E/A/E), respectively, (Fig. 1). The 212-bp sequence towards the C-terminal region of MSP-3, corresponding to nucleotide positions 571 and 783, were monomorphic in all isolates.

Furthermore, when the 17 nucleotide sequences with the 3D7 allele (H1 and H2) of *msp*-*3* were analysed separately, only one SNP locus, at nucleotide position 307 (GCT/TCT), was detected between the two haplotypes. This point mutation resulted in a non-synonymous amino acid change at amino acid position 103 (A/S), demonstrating the relatively low genetic diversity of *msp*-*3* among 3D7 alleles. In contrast, a separate analysis of the 42 sequences with the K1 allele (H3, H4, H5, 6A, H6B, H7) revealed 51 SNP loci (excluding gaps), resulting in 40 non-synonymous mutations and seven synonymous mutations. This result, therefore, clearly depicts the different levels of genetic diversity between the K1 and 3D7 *msp*-*3* alleles.

Because of the observed high level of polymorphism, a next goal was to determine whether natural selection contributes to the diversity of this gene. The *d*_*N*_/*d*_*S*_ ratio was calculated using the sequences of the 59 samples as described in Methods. When the nucleotide sequences encoding the AHR region (nucleotide positions 232–570) of MSP-3 were analysed, the *d*_*N*_/*d*_*S*_ ratio was 1.08 (greater than 1) (*P* = 0.818), although the value did not show a significant positive departure from zero. Thus, there was a tendency for positive diversifying selection. Furthermore, the three within-population tests for neutrality (Tajima’s *D*, Fu and Li’s D* and F* tests) were all significant, with values of 2.611 (*P* < 0.01), 1.998 (*P* < 0.02) and 2.644 (*P* < 0.02), respectively. To determine whether specific region(s) of the AHR region was under selection, a sliding window plot analysis was performed. The values of *D*, D* and F* were calculated on a window of 60 bp moving in steps of three sites. As shown in Fig. 2, the polymorphisms between the nucleotide positions 252–528, excluding monomorphic nucleotide positions 351–375, showed significant departure from neutrality in all three tests. For comparison, sliding window plots of nucleotide diversity (π), Tajima’s *D* test, Fu and Li’s D* test and Fu and Li’s F* test a window size of 100 bases and a step size of 25 bases (default setting) were also analysed [48]. Regions of *msp*-*3* with significant values from the sliding window plots with a window size of 100 bases and a step size of 25 bases and a window size of 60 bases and a step size of 3 bases were largely overlapped (see Additional file 2). The alternation of a window size and a step size of sliding window plots did not affect the conclusion. Altogether, the comparison of the non-synonymous and synonymous mutations, *D*, D* and F* all suggested that the polymorphisms in the AHR region of MSP-3 were maintained by positive diversifying selection, which was in agreement with the previous neutrality test analysis using the *msp*-*3* sequences from Thailand and Nigeria [37].

### Amino acid sequence analysis

**Fig. 4:**
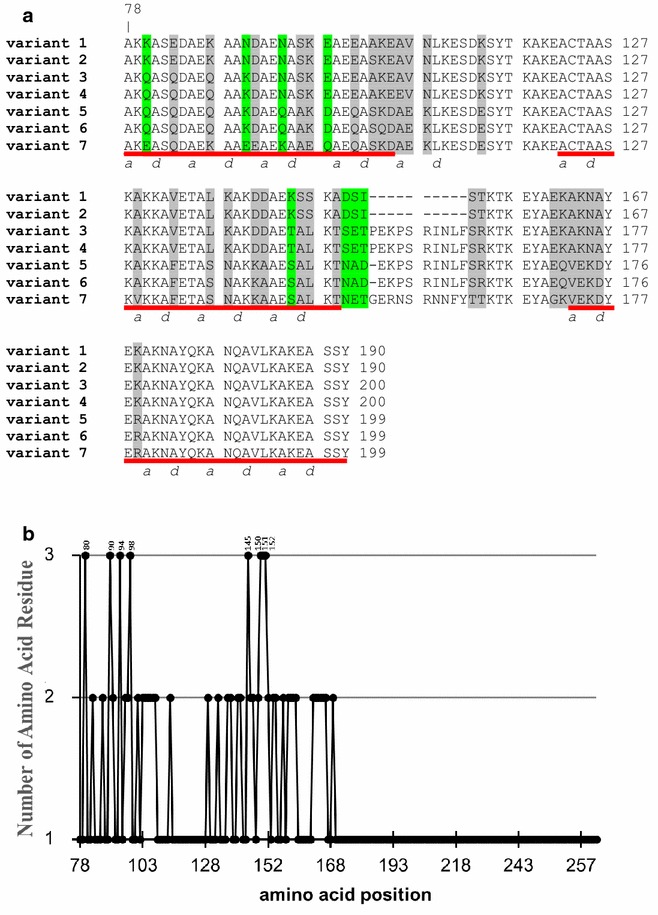
Partial amino acid sequences of *Plasmodium falciparum* MSP-3. **a** Alignment of the deduced amino acid sequences encoding the AHRs, revealing seven sequence variants. Dimorphic and trimorphic amino acid residues are highlighted in *grey* and *green*, respectively. Variants 1 and 7 are identical to the MSP-3 sequences of the *P. falciparum* reference strains 3D7 and K1, respectively. Two haplotypes (H1 and H2) of the 3D7 allele encode variants 1 and 2, while the other five haplotypes encode variants 3–7. Note that haplotypes 6A and 6B encode the same amino acid sequence (variant 6). **b** Location of the polymorphic amino acid residues in MSP-3 sequences. The positions of MSP-3 sequences are numbered after *P. falciparum* strain 3D7

The deduced amino acid sequences of *P. falciparum* MSP-3 were translated from the DNA sequences of the eight haplotypes of the 59 nucleotide sequences. The translated sequences represent the three blocks of the AHR regions, covering amino acid positions 78–190 of the reference strain 3D7. Each DNA haplotype of *msp*-*3* produced a unique amino acid sequence, except for haplotypes 6A and 6B that generated the same amino acid sequences and so seven amino acid sequence variants were found (Table 1). There were two amino acid sequence variants (variants 1 and 2) for two haplotypes of the 3D7 allele, and five amino acid sequence variants (variants 3–7) for six haplotypes of the K1 allele (Fig. 4a).

**Table 1:** Summary of the pairwise *F*_*st*_ values of MSP-3 variants between *Plasmodium falciparum* populations in Thailand

Location	Trat	Kanchanaburi	Mae Hong Son	Ubon Ratchathani	Ranong
Kanchanaburi	0.04492 (*P* = 0.17)				
Mae Hong Son	0.0645 (*P* = 0.92)	0.04634 (*P* = 0.17)			
Ubon Ratchathani	0.18483* (*P* = 0.02)	0.09703 (*P* = 0.08)	0.26705** (*P* = 0.00)		
Ranong	0.08137 (*P* = 0.09)	0.02499 (*P* = 0.26)	0.05051 (*P* = 0.19)	0.28465** (*P* = 0.00)	
Tak	0.08493 (*P* = 0.06)	0.0086 (*P* = 0.30)	0.06375 (*P* = 0.07)	0.20385** (*P* = 0.00)	0.03830 (*P* = 0.92)

Data from Tak province is from [37]

Asterisks indicate the significant *F*_*st*_ values (sign of population differentiation)

* *P* < 0.05

** *P* < 0.01

The amino sequence analysis showed a total of 45 amino acid changes across the 59 sequences. Of these polymorphic sites, 37 were dimorphic and eight were trimorphic. In order to determine the distribution of amino acid polymorphisms across the AHR region of the MSP-3 sequence, the location of substitutions and the number of amino acid types observed at each site were plotted. As shown in Fig. 4b, the amino acid polymorphisms were predominantly clustered between amino acid positions 80 and 169, located between block 1 and the first heptad repeat of block 3. Block 1 of the AHR region also contained the highest number of polymorphic sites, four of which were trimorphic amino acid substitutions. The other trimorphic sites were clustered at the non-repetitive sequence between blocks 2 and 3.

Despite this diversity, block 1 in the seven MSP-3 variants also contained a fifth AHR based on the ‘AXXAXXX’ motif except with leucine (L), rather than alanine (A) in the *d* position (Fig. 4a), similar to other reports [32, 49]. In addition, blocks 2 and 3 of the AHR region in the seven variants contained four AHRs except for block 2 of variants 1, 2 and 7, and block 3 of variants 5, 6 and 7, which contained only three AHRs.

### Distribution of MSP-3 variants in *Plasmodium falciparum* in Thailand

**Fig. 5:**
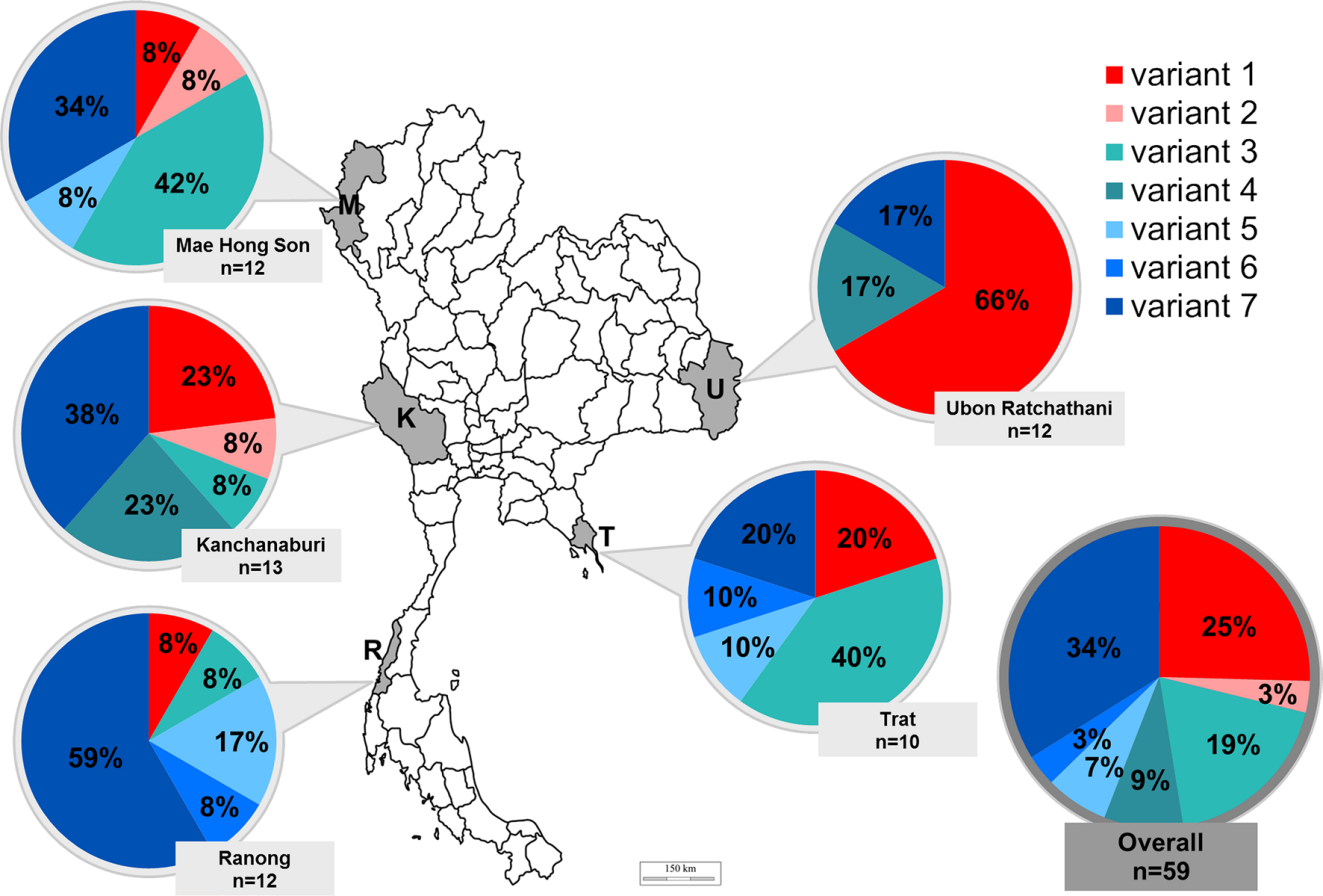
Frequency and distribution of MSP-3 variants in Thai populations of *Plasmodium falciparum. Plasmodium falciparum* samples were collected from endemic regions of Thailand: Mae Hong Son (MH), Kanchanaburi (K), Ranong (R), Trat (T), and Ubon Ratchatani (U). Ubon Ratchathani is located at the Laos–Thailand border, Trat is located at the Cambodian–Thailand border, and the other three sites are located along the Myanmar–Thailand border. Numbers (n) of the total parasite isolates in Thailand (overall) or the parasites from each locality are displayed in the *bracket*. Numbers in *pie charts* represent percentage of MSP-3 variants. Variants 1 and 2 are from the 3D7 allele of *msp*-*3*, while the others belong to the K1 allele of *msp*-*3*

The overall prevalence and distribution patterns of the seven MSP-3 variants in *P. falciparum* populations in Thailand are shown in Fig. 5. The two most prevalent types, variants 1 and 7, co-existed in all sampled sites, although the proportions of these variants varied remarkably in the different geographical locations of the parasites. Variant 1 was the most prevalent MSP-3 type in the Ubon Ratchatani population, at a frequency of 66 %, whereas variant 7 was the most prevalent type in the Ranong and Kanchanaburi populations, at a frequency of 59 and 38 %, respectively. Interestingly, in the *P. falciparum* populations in Mae Hong Son and Trat, variant 3 appeared to be more prevalent than variants 7 and 1, with a frequency of 42 and 40 %, respectively. The other variants (2–5 and 6) were, however, minor variants, each of which was represented at less than 10 % in each population.

In addition, Fig. 5 also summarizes the different levels of genetic diversity in the *P. falciparum* populations based on the MSP-3 variants. Those in Mae Hong Son, Kanchanaburi, Ranong, and Trat had five different variants. In contrast, only three variants were present in the Ubon Ratchatani population suggesting that the genetic diversity of *P. falciparum* in the Thai–Laos border was lower than in the Thai–Myanmar and Thai–Cambodia borders.

In order to compare patterns of distribution of MSP-3 variants between *P. falciparum* populations in Thailand, pair-wise inter-population comparison were conducted for each population using Wright’s Fixation index (*F*_*st*_). The *F*_*st*_ values were significant (*P* < 0.05) between Ubon Ratchatani and the Trat, Ranong and Mae Hong Son populations, but not between those at Ubon Ratchatani and Kanchanaburi (Table 1). This indicates potential population sub-division between *P. falciparum* populations in Ubon Ratchatani and the other regions in Thailand, excluding Kanchanaburi due to their sharing of the same three variants (1, 4 and 7) (Fig. 4). The distribution of MSP-3 patterns between the four populations in Mae Hong Son, Kanchanaburi, Ranong, and Trat showed low and non-significant F_st_ values, indicating a similar distribution of the MSP-3 variants between these four *P. falciparum* populations.

### Comparison of MSP-3 variants in the *Plasmodium falciparum* reference strains and natural isolates

The nucleotide sequences of the *msp*-*3* gene from 14 laboratory clones from the *P. falciparum* genome database were retrieved from the National Center for Biotechnology Information (NCBI) website (see Additional file 3). The analysis showed that seven of these strains (HB3 from Honduras, RO-33 from Ghana, 7G8 from Brazil, Santa Lucina from El Salvador, RAJ116 from India, MaliPS096_E11 from Mali, NF54 of unknown origin) had variant 1 of MSP-3, similar to that of *P. falciparum* strain 3D7. The other seven strains (2,000,708 from Tanzania, NF135/5.C10 from Cambodia, VS/1 and Vietnam Oak-Knoll from Vietnam, Dd2 from Laos, IGH-CR4 from India, FCH/4 from Philippines) carried variants 3, 4, 6 and 7 of MSP-3, all of which were derived from the K1 allele. The results demonstrate the diversity of MSP-3 variants in laboratory clones.

In addition, the *msp*-*3* sequences of natural isolates of *P. falciparum* in Thailand, India and Nigeria were obtained through the literature search at the NCBI and Scopus websites (Additional file 4) [37]. The data showed that *P. falciparum* from Tak province (n = 50) had the same seven MSP-3 sequence variants as in this study of Thai populations, and so all these variants were re-discovered and confirmed in the present study. The *Fst* analysis also showed that the *P. falciparum* population from Tak province was similar to *P. falciparum* in other regions in Thailand, except for Ubon Ratchanani, in which the 3D7 allele was the dominant type (Table 1). In addition, the *P. falciparum* population in India (n = 56) had five of the seven MSP-3 variants found in Thailand. This broadly similar distribution pattern of MSP-3 variants between *P. falciparum* populations in South Asia and Southeast Asia supports the existence of gene flow between these parasite populations or that the parasite populations are closely genetically related. In contrast, nine variants of MSP-3 were identified in *P. falciparum* populations in Nigeria (n = 51), of which five (variants 8–12, see Additional file 5) were distributed exclusively in the African population, suggesting that *P. falciparum* in Africa were genetically distinct from those in Asia. The population differentiation between *P. falciparum* populations in Asia and Africa was also supported by the *Fst* analysis (Table 2).

**Table 2:** Summary of pairwise *F*_*st*_ values of MSP-3 variants between *Plasmodium falciparum* populations in Thailand, India and Nigeria

	Thailand^a^	India
India^b^	0.00983 (*P* = 0.17)	
Nigeria^c^	0.10673** (*P* = 0.00)	0.14867** (*P* = 0.00)

** Indicate significant (*P* < 0.01) *F*_*st*_ values (sign of population differentiation)

^a^Sequence data from Thailand included the data generated in the present work only [37]

^b^Sequence data from India was retrieved from the NCBI nucleotide database (see Additional files for details)

^c^Sequence data from Nigeria was obtained from Polley et al. [37]

## Discussion

The MSP-3 of the human malaria parasite *P. falciparum* is a key surface antigen and is considered a candidate antigen for the development of a blood-stage malaria vaccine. While the *msp*-*3* gene sequences of laboratory strains of *P. falciparum* are extremely useful for the development of current MSP-3 vaccines [17, 22], the study of *msp*-*3* sequences in natural parasite populations can be applicable for monitoring the vaccine efficacy and designing relevant local policies for malaria control. A previous, preliminary, cross-sectional survey of the *msp*-3 allelic diversity in natural *P. falciparum* populations in Thailand demonstrated the biased distribution of the K1 allele in four parasite populations at the Thailand–Myanmar and Thailand–Cambodia borders, while the 3D7 allele of *msp*-*3* was more prevalent in a population at the Thailand–Laos border [28]. This raises the questions as to how and why the population structure of *P. falciparum* at the Thailand–Laos border is genetically distinct from other regions in Thailand. The present study aimed to investigate the sequence diversity of the *msp*-*3* gene in *P. falciparum* populations in Thailand.

The main finding of this work was that eight DNA haplotypes of the *msp*-3 gene were detected, with two and six haplotypes representing the 3D7 and K1 allelic types, respectively. Although all the haplotypes had previously been detected in *P. falciparum* samples from the Tak province in western Thailand [37], the results from the present work confirmed the existence of sequence polymorphism in the *P. falciparum msp*-*3* gene across diverse *P. falciparum* populations in Thailand. The alignment of the nucleotide sequences of *msp*-*3* also revealed sets of dimorphic SNPs and indel mutations, defining the 3D7 and K1 allelic types, and was supported by the ML-based phylogenetic analysis. The majority of the dimorphic sites were predominantly clustered in the N-terminal extremity within and flanking the AHRs, consistent with previous reports [32, 37], and supporting the view that the *msp*-*3* polymorphism in laboratory strains and natural isolates is highly restricted. The sequence analysis also showed a lack of genetic recombination between the K1 and 3D7 allelic types or alternative polymorphisms in natural *P. falciparum* populations in Thailand, although novel alleles of *msp*-*3* were detected in Iran and African countries at a very low frequency [33, 35]. The lack of recombination between the two allelic types is likely due to the high divergence of the dimorphic sequence that then prevents crossing over at meiosis. Alternatively, the *msp*-3 sequence dimorphism may be maintained in *P. falciparum* by balancing selection that has prevented the loss of divergent alleles over a long period of time [29, 50].

The significantly elevated Tajima’s *D*, Fu and Li’ D*, Fu and Li’ F* values indicated that the Thai populations of *P. falciparum* exhibit a significant departure from neutral equilibrium expectations for the *msp*-*3* gene. This result was consistent with the previous analysis using the *msp*-*3* sequences from Tak province, Thailand [37]. Sliding window plot analysis of the neutrality tests also showed the AHRs of *msp*-*3* accumulated a significantly higher rate of mutations and such polymorphism was likely to be maintained by frequency-dependent selection, presumably caused by protective immunity. This is in agreement with previous immunological data that the N-terminal region of MSP-3 contains epitopes that induce allelic-specific protective antibodies, or represent the sites of antigenic diversity among MSP-3 polypeptides [13, 26, 51, 52]. It is also interesting to note that despite the diversity within the N-terminal domains, the copy number and repeats of the ‘AXXAXXX’ motif is highly conserved. This reflects the necessity to maintain the residue contact that is critical for the α-helical secondary structure formation [9].

In contrast to the polymorphic N-terminal region, the C-terminal region of MSP-3 is relatively conserved among the various *P. falciparum* isolates in Thailand. The most probable explanation for the limited diversity at the AHR franking region is the functional constraint of the protein. This part of the protein facilitates the oligomerization of the proteins, which is essential for the binding of haem released from unprocessed haemoglobin during parasite egression [6, 53]. Alternatively, this region of the protein may play an important role in the invasion of the host erythrocyte, as truncation of MSP-3 to remove the leucine zipper but to retain the AHR and glutamic-rich regions led to inference with normal trafficking of MSP-3, and the transgenic parasites were less efficient at invading host erythrocytes [11].

It has been shown that other malaria parasite antigens also exhibited dimorphism, such as MSP-1 and MSP-2 [54–56]. There have been reports of statistically significant differences in the proportions of alleles at the *msp*-*1* and *msp*-*2* loci between mild and severe malaria cases [57–61]. However, in a genetic analysis of *P. falciparum msp*-*3* in Peru correlation between the *msp*-*3* alleles and symptom status was not detected [62]. It would be of interest to investigate whether the dimorphism in *msp*-*3* locus was also associated with severe or mild malaria in Thailand and other endemic regions. Possible disease associations may need to be tested in further studies by analyzing parasite alleles alongside measurements of clinical status of patients and allele-specific immune responses in the same subjects.

In addition to the allelic dimorphism, polymorphisms were also detected within each of the K1 and 3D7 allelic types. A large number of polymorphic sites and indel mutations were detected among six haplotypes of the K1 alleles, while only a dimorphic site was found between two haplotypes of the 3D7 alleles. This indicates that the levels of genetic diversity within the K1-allelic type are much higher than those of the 3D7-allelic sequences, and is probably due to the greater prevalence of the K1 allele in parasite populations in Thailand. Interestingly, in *P. falciparum* population in Peru, the majority of the parasites (~90 %) possessed the 3D7 allele [62]. The study showed that about 1.8 % of the samples with the 3D7 allele (n = 570) had the non-synonymous mutation at nucleotide position 203 (C203T), indicating the low genetic diversity in the 3D7 allele of *msp*-*3*. These results may support the view that the 3D7 allele of *msp*-*3* originates from introgression from another *Plasmodium* species, or it recently emerged from the K1 allele, or a recent introduction of 3D7 alleles to the endemic regions [29, 37, 62].

The sequence analysis presented here may be useful in predicting target sites in MSP-3 for protective immunity. The data presented here suggest that the AHR in the N-terminal region of MSP-3 contains allelic-specific epitopes that are immunogenic than the conserved sequences. This notion is supported by the clinical immunological analysis of anti-MSP-3 antibodies in Gambian children, which showed a bias in reactivity towards either the K1 or 3D7 type recombinant protein [37], suggesting that allele-specific epitopes in MSP-3 may induce stronger antibody responses than the conserved epitopes. Similarly, in cohort studies in Kenya and Peru, antibodies responses to allelic specific epitopes of MSP-3 were more common than those to a conserved C-terminal region of MSP-3 [13, 51]. Interestingly, these studies also revealed that allelic-specific antibody responses to MSP-3 were associated with a lower risk of clinical malaria episodes [13, 37]. However, the leucine zipper-like domain at the C-terminus of MSP-3 was excluded in this analysis and so it is possible that this domain was the target of protective immunity. Indeed, it has been reported that antibodies to the conserved C-terminal region of MSP-3 were also associated with clinical protection [27, 63]. Overall, these studies suggest that the N- and C-terminal regions of MSP-3 are targets of host protective immunity, and either or both parts of them or the full-length MSP-3 may be used as vaccine candidates. Combining the effect of allele-specific immunity together with that induced to the conserved C-terminal region of MSP-3 gene may provide a vaccine of long-term usefulness.

Last but not least, the sequence analysis of the *msp*-*3* gene could infer the population structure of *P. falciparum* in Thailand, where the distribution pattern of MSP-3 variants depicts a differential genetic diversity among the populations in Thailand. In contrast to a previous study, in which *msp*-*3* alleles were classified into two types by PCR [34], the sequence information of *msp*-*3* showed that there were as many as five variants of MSP-3 in all of the parasite populations examined except for that in Ubon Ratchatani. This result indicates that the levels of genetic diversity of *P. falciparum* near the Thailand–Myanmar and Thailand–Cambodia borders were greater than that at the Thailand–Laos border. The *F*_*st*_ index revealed that the *P. falciparum* population structure in Ubon Ratchatani was similar to that at Kanchanaburi, since the two populations shared the same three MSP-3 variants. This finding may suggest the origin and gene flow between the parasite populations in Thailand. The *F*_*st*_ analysis of MSP-3 variants in Thailand, India and Nigeria also indicated that *P. falciparum* populations in Thailand and India were more closely related than those parasites in Nigeria. This finding was also supported by the previous genome-wide studies that demonstrated population subdivision by continent in *P. falciparum* natural populations [4, 64]. The distribution map of the MSP-3 variants also showed that variants 1 of the 3D7 type and 7 of the K1 type were the most abundant types. In fact, these two variants were also present in *P. falciparum* populations in India and Nigeria, despite different geographical origins. If the full-length MSP-3 was developed as a vaccine, it would be beneficial to include both allelic forms, i.e., variants 1 and 7 as the representative allele of the 3D7 and K1 types in a vaccine formulation. Such a vaccine might improve the efficacy against clinical malaria without increasing the selection pressure on the locus.

## Conclusion

This study extends the understanding of genetic variation in the MSP-3 protein and prevalence of MSP-3 alleles in natural populations of *P. falciparum* in Thailand. The nucleotide sequence analysis of *msp*-*3* revealed that the polymorphisms that define allelic classes of *msp*-*3* are attributed to the indel and SNPs in the gene. The sequence information also aids in identifying regions under positive selection, thereby providing knowledge for a better design of any MSP-3-based malaria vaccine, as well as revealing the population structure of *P. falciparum* across Thailand, which is of use for monitoring and control of this parasite.

## Additional files

**Additional file 1.** Alleles and variants of merozoite surface protein-3 in Plasmodium falciparum isolates in Thailand.

**Additional file 2.** Sliding window plots of the average pairwise nucleotide diversity (π), Tajima’s *D* values, Fu and Li’s *D** values and Fu and Li’s *F** values of the *msp-3* gene of *P. falciparum* in Thailand.

**Additional file 3.** Variants of the MSP-3 protein sequence in reference strains of *Plasmodium falciparum*.

**Additional file 4.**Frequency of MSP-3 variants and nucleotide sequence ID of the *msp-3* gene of *P. falciparum* in Thailand, Nigeria and India.

**Additional file 5.** Maximum-likelihood tree of *msp-3* gene from *P. falciparum* populations in Thailand and Nigeria.
