# Supplementary material for: Genetic diversity of the merozoite surface protein-3 gene in Plasmodium falciparum populations in Thailand
Source: Malar J. 2016 Oct 21;15:517. doi: 10.1186/s12936-016-1566-1 (PMC5073822; doi:10.1186/s12936-016-1566-1)
Supplement: Supplementary file 1 — Additional file 1. Alleles and variants of merozoite surface protein-3 in Plasmodium falciparum isolates in Thailand. [file 12936_2016_1566_MOESM1_ESM.doc]

**Additional file 1 -** **Alleles and variants of merozoite surface protein-3 in *Plasmodium falciparum* isolates in Thailand.** Alleles of *msp-3* were classified according to size after PCR amplification and agarose gel electrophoresis. Nucleotide sequence alignments revealed eight unique haplotypes (H1–5, H6A, H6B and H7), generating seven amino acid sequence variants (1–7) since haplotypes H6A and H6B generated identical amino acid sequences.

| **Allele** | **Haplotype** | **Variant of MSP-3** | **Parasite isolate^a^** | **Number (%)** |
| --- | --- | --- | --- | --- |
| 3D7 | H1 | 1 (3D7)* | K74, K391, K389, RN133, TD504, TD508, UB27, UB7, UB28, UB59, UB52, UB51, UB58, UB50, MH32 | 15 (25%) |
|  | H2 | 2 | K195, MH11 | 2 (3%) |
| K1 | H3 | 3 | K215, TD531, TD533, TD554, TD556, MH50, MH51, MH61, MH65, MH65, RN28 | 11 (19%) |
|  | H4 | 4 | K64, K185, K392, UB82, UB84 | 5 (9%) |
|  | H5 | 5 | RN68, RN130, TD510, MH18 | 4 (7%) |
|  | H6A | 6 | RN131 | 1 (1.5%) |
|  | H6B |  | TD515 | 1 (1.5%) |
|  | H7 | 7 (K1)* | K58, K165, K205, K397, K386, RN19, RN31, RN26, RN36, RN72, RN66, RN129, TD530, TD529, UB14, UB85, MH06, MH10, MH20, MH24 | 20 (34%) |

The *msp-3* sequences of the 3D7 and K1 strains match haplotypes H1 and H7, respectively.

^a^Name of parasite isolates indicates the origin of the parasites: MH, Mae Hong Son; K, Kanchanaburi; RN, Ranong; TD, Trat; UB, Ubon Ratchatani.
