# Supplementary material for: Genetic diversity of the merozoite surface protein-3 gene in Plasmodium falciparum populations in Thailand
Source: Malar J. 2016 Oct 21;15:517. doi: 10.1186/s12936-016-1566-1 (PMC5073822; doi:10.1186/s12936-016-1566-1)
Supplement: Supplementary file 2 — Additional file 2. Sliding window plots of the average pairwise nucleotide diversity (π), Tajima’s D values, Fu and Li’s D* values and Fu and Li’s F* values of the msp-3 gene of P. falciparum in Thailand. [file 12936_2016_1566_MOESM2_ESM.doc]

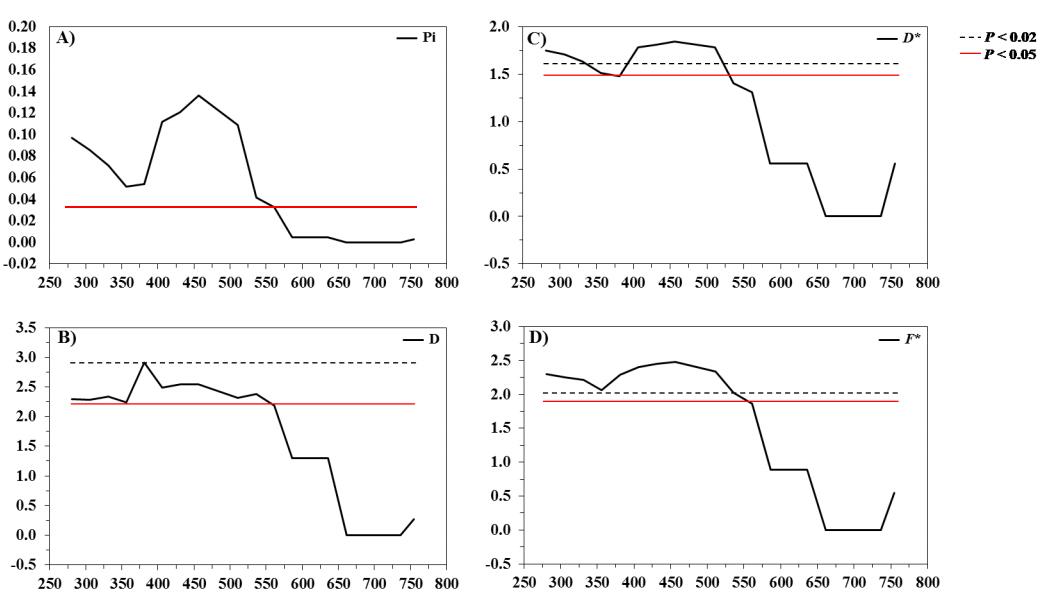


**Additional file 2 - Sliding window plots of the average pairwise nucleotide diversity (π), Tajima’s *D* values, Fu and Li’s *D** values and Fu and Li’s *F** values of the *msp-3* gene of *P. falciparum* in Thailand.** (A) The nucleotide diversity was plotted by a sliding window with a window size of 100 and a step size of 25 (default setting). The maximum diversity (**π** > 0.03) was detected between nucleotide positions 252 and 561. Sliding window plots of (B) Tajima’s *D* values, (C) Fu and Li’s *D** values and (D) Fu and Li’s *F** values with a window size of 100 nucleotides and a step size of 25 nucleotides detected significant values at nucleotide positions 252 - 552, 252 – 528 and 252 - 561 bp., respectively.
