# Supplementary material for: Genetic diversity of the merozoite surface protein-3 gene in Plasmodium falciparum populations in Thailand
Source: Malar J. 2016 Oct 21;15:517. doi: 10.1186/s12936-016-1566-1 (PMC5073822; doi:10.1186/s12936-016-1566-1)
Supplement: Supplementary file 3 — Additional file 3. Variants of the MSP-3 protein sequence in reference strains of Plasmodium falciparum. [file 12936_2016_1566_MOESM3_ESM.doc]

**Additional file 3 - Variants of the MSP-3 protein sequence in reference strains of *Plasmodium falciparum*. Draft genome sequences of 16 common laboratory strains of *P. falciparum* were retrieved from the NCBI genome database (Genome Assembly)** [**http://www.ncbi.nlm.nih.gov/assembly**](http://www.ncbi.nlm.nih.gov/assembly)**. The *msp-3* sequences were translated and classified according to the amino acid sequence variants (see Figure 4). Grey and white backgrounds indicate the 3D7 and K1 alleles of *msp-3*, respectively.**

| Name | Variant | Origin  (continent) | Sequence ID |
| --- | --- | --- | --- |
| **3D7** | 1 | Clones of NF54 | GCA_000002765.1 |
| HB3 | 1 | Honduras  (Central America) | GCA_000149665.2 |
| RO-33 | 1 | Ghana  (African) | GCA_000150335.1 |
| 7G8 | 1 | Brazil  (South America) | GCA_000150435.3 |
| Santa Lucia | 1 | El Salvador  (South America) | GCA_000150455.3 |
| RAJ116 | 1 | India  (South Asia) | GCA_000186025.2 |
| NF54 | 1 | Patient in Amsterdam  (unknown origin) | GCA_000401695.2 |
| MaliPS096_E11 | 1 | Mali  (Africa) | GCA_000521035.1 |
| K1 | 7 | Thailand  (Southeast Asia) | GCA_000150355.1 |
| Tanzania (2000708) | 3 | Tanzania  (Africa) | GCA_000521055.1 |
| NF135/5.C10 | 4 | Cambodia  (Southeast Asia) | GCA_000521075.1 |
| VS/1 | 6 | Vietnam  (Southeast Asia) | GCA_000150295.1 |
| Dd2 | 7 | Indochina  (Southeast Asia) | GCA_000149795.1 |
| IGH-CR14 | 7 | India  (South Asia) | GCA_000186055.2 |
| Vietnam Oak-Knoll | 7 | Vietnam  (Southeast Asia) | GCA_000521015.1 |
| FCH/4 | 7 | Philippines  (Southeast Asia) | GCA 000521155 1 |
