# Supplementary material for: Genetic diversity of the merozoite surface protein-3 gene in Plasmodium falciparum populations in Thailand
Source: Malar J. 2016 Oct 21;15:517. doi: 10.1186/s12936-016-1566-1 (PMC5073822; doi:10.1186/s12936-016-1566-1)
Supplement: Supplementary file 4 — Additional file 4.Frequency of MSP-3 variants and nucleotide sequence ID of the msp-3 gene of P. falciparum in Thailand, Nigeria and India. [file 12936_2016_1566_MOESM4_ESM.doc]

**Additional file 4.** **Frequency of MSP-3 variants and nucleotide sequence ID of the *msp-3* gene of *P. falciparum* in Thailand, Nigeria and India.**

**Table 4A - Frequency of variants of merozoite surface protein-3 in natural populations of *Plasmodium falciparum* in Thailand, Nigeria and India.**

| **Variant** |  | | **Frequency of MSP-3 variants** | | | **Allele** |
| --- | --- | --- | --- | --- | --- | --- |
|  | **Thailand**  **(Present study)** | **Thailand**  **(Tak province)^a^** | | **Nigeria^a^** | **India^b^** |  |
| 1 | 25.4% | 18.0% | | 58.8% | 17.9% | 3D7 |
| 2 | 3.4% | 2.0% | | - | - | 3D7 |
| 3 | 18.6% | 8.0% | | 2.0% | 26.8% | K1 |
| 4 | 8.5% | 4.0% | | - | - | K1 |
| 5 | 6.8% | 14.0% | | 7.8% | 14.3% | K1 |
| 6 | 3.4% | 2.0% | | - | 14.3% | K1 |
| 7 | 33.9% | 52.0% | | 13.7% | 26.8% | K1 |
| 8 | - | - | | 3.9% | - | 3D7 |
| 9 | - | - | | 2.0% | - | K1 |
| 10 | - | - | | 3.9% | - | K1 |
| 11 | - | - | | 5.9% | - | K1 |
| 12 | - | - | | 2.0% | - | K1 |

^a^The *msp-3* sequences of *P. falciparum* populations in Tak province, Thailand and in Nigeria are obtained from Polley *et al.* [37].

^b^Sequences of *P. falciparum* population in India are retrieved from the NCBI nucleotide databases, available at the website: [http://www.ncbi.nlm.nih.gov](http://www.ncbi.nlm.nih.gov/).

More detailed lists of the *msp-3* sequence ID from Tak province, Nigeria and India are shown in Tables 4B, 4C and 4D, respectively.

**Table 4B – Nucleotide sequence ID of the *msp-3* sequences from *P. falciparum* in Tak province, Thailand [37].** (n = 50)

| **No** | **GI number** | **Accession number** | **variant** |  | **No** | **GI number** | **Accession number** | **variant** |
| --- | --- | --- | --- | --- | --- | --- | --- | --- |
| 1 | 113207087 | AM161544.1 | 7 |  | 26 | 113207137 | AM161569.1 | 7 |
| 2 | 113207089 | AM161545.1 | 7 |  | 27 | 113207139 | AM161570.1 | 5 |
| 3 | 113207091 | AM161546.1 | 7 |  | 28 | 113207141 | AM161571.1 | 5 |
| 4 | 113207093 | AM161547.1 | 7 |  | 29 | 113207143 | AM161572.1 | 5 |
| 5 | 113207095 | AM161548.1 | 7 |  | 30 | 113207145 | AM161573.1 | 5 |
| 6 | 113207097 | AM161549.1 | 7 |  | 31 | 113207147 | AM161574.1 | 5 |
| 7 | 113207099 | AM161550.1 | 7 |  | 32 | 113207149 | AM161575.1 | 5 |
| 8 | 113207101 | AM161551.1 | 7 |  | 33 | 113207151 | AM161576.1 | 5 |
| 9 | 113207103 | AM161552.1 | 7 |  | 34 | 113207153 | AM161577.1 | 6 |
| 10 | 113207105 | AM161553.1 | 7 |  | 35 | 113207155 | AM161578.1 | 4 |
| 11 | 113207107 | AM161554.1 | 7 |  | 36 | 113207157 | AM161579.1 | 4 |
| 12 | 113207109 | AM161555.1 | 7 |  | 37 | 113207159 | AM161580.1 | 3 |
| 13 | 113207111 | AM161556.1 | 7 |  | 38 | 113207161 | AM161581.1 | 3 |
| 14 | 113207113 | AM161557.1 | 7 |  | 39 | 113207163 | AM161582.1 | 3 |
| 15 | 113207115 | AM161558.1 | 7 |  | 40 | 113207165 | AM161583.1 | 3 |
| 16 | 113207117 | AM161559.1 | 7 |  | 41 | 113207167 | AM161584.1 | 1 |
| 17 | 113207119 | AM161560.1 | 7 |  | 42 | 113207169 | AM161585.1 | 1 |
| 18 | 113207121 | AM161561.1 | 7 |  | 43 | 113207171 | AM161586.1 | 1 |
| 19 | 113207123 | AM161562.1 | 7 |  | 44 | 113207173 | AM161587.1 | 1 |
| 20 | 113207125 | AM161563.1 | 7 |  | 45 | 113207175 | AM161588.1 | 1 |
| 21 | 113207127 | AM161564.1 | 7 |  | 46 | 113207177 | AM161589.1 | 1 |
| 22 | 113207129 | AM161565.1 | 7 |  | 47 | 113207179 | AM161590.1 | 1 |
| 23 | 113207131 | AM161566.1 | 7 |  | 48 | 113207181 | AM161591.1 | 1 |
| 24 | 113207133 | AM161567.1 | 7 |  | 49 | 113207183 | AM161592.1 | 1 |
| 25 | 113207135 | AM161568.1 | 7 |  | 50 | 113207185 | AM161593.1 | 2 |

**Table 4C – Nucleotide sequence ID of the *msp-3* sequences from *P. falciparum* in Nigeria [37].** (n = 51)

| **No** | **GI number** | **Accession number** | **variant** |  | **No** | **GI number** | **Accession number** | **variant** |
| --- | --- | --- | --- | --- | --- | --- | --- | --- |
| 1 | 113207187 | AM161594.1 | 10 |  | 27 | 113207239 | AM161620.1 | 1 |
| 2 | 113207189 | AM161595.1 | 9 |  | 28 | 113207241 | AM161621.1 | 1 |
| 3 | 113207191 | AM161596.1 | 7 |  | 29 | 113207243 | AM161622.1 | 1 |
| 4 | 113207193 | AM161597.1 | 7 |  | 30 | 113207245 | AM161623.1 | 1 |
| 5 | 113207195 | AM161598.1 | 7 |  | 31 | 113207247 | AM161624.1 | 1 |
| 6 | 113207197 | AM161599.1 | 7 |  | 32 | 113207249 | AM161625.1 | 1 |
| 7 | 113207199 | AM161600.1 | 7 |  | 33 | 113207251 | AM161626.1 | 1 |
| 8 | 113207201 | AM161601.1 | 7 |  | 34 | 113207253 | AM161627.1 | 1 |
| 9 | 113207203 | AM161602.1 | 7 |  | 35 | 113207255 | AM161628.1 | 1 |
| 10 | 113207205 | AM161603.1 | 12 |  | 36 | 113207257 | AM161629.1 | 1 |
| 11 | 113207207 | AM161604.1 | 7 |  | 37 | 113207259 | AM161630.1 | 1 |
| 12 | 113207209 | AM161605.1 | 5 |  | 38 | 113207261 | AM161631.1 | 1 |
| 13 | 113207211 | AM161606.1 | 5 |  | 39 | 113207263 | AM161632.1 | 1 |
| 14 | 113207213 | AM161607.1 | 5 |  | 40 | 113207265 | AM161633.1 | 1 |
| 15 | 113207215 | AM161608.1 | 5 |  | 41 | 113207267 | AM161634.1 | 1 |
| 16 | 113207217 | AM161609.1 | 11 |  | 42 | 113207269 | AM161635.1 | 1 |
| 17 | 113207219 | AM161610.1 | 11 |  | 43 | 113207271 | AM161636.1 | 1 |
| 18 | 113207221 | AM161611.1 | 11 |  | 44 | 113207273 | AM161637.1 | 1 |
| 19 | 113207223 | AM161612.1 | 3 |  | 45 | 113207275 | AM161638.1 | 1 |
| 20 | 113207225 | AM161613.1 | 1 |  | 46 | 113207277 | AM161639.1 | 1 |
| 21 | 113207227 | AM161614.1 | 1 |  | 47 | 113207279 | AM161640.1 | 1 |
| 22 | 113207229 | AM161615.1 | 1 |  | 48 | 113207281 | AM161641.1 | 1 |
| 23 | 113207231 | AM161616.1 | 8 |  | 49 | 113207283 | AM161642.1 | 1 |
| 24 | 113207233 | AM161617.1 | 8 |  | 50 | 113207285 | AM161643.1 | 1 |
| 25 | 113207235 | AM161618.1 | 1 |  | 51 | 113207287 | AM161644.1 | 1 |
| 26 | 113207237 | AM161619.1 | 1 |  |  |  |  |  |

**Table 4D – Nucleotide sequence ID of the *msp-3* sequences from *P. falciparum* in India (unpublish data).** (n = 56)

| **No** | **GI number** | **Accession number** | **variant** |  | **No** | **GI number** | **Accession number** | **variant** |
| --- | --- | --- | --- | --- | --- | --- | --- | --- |
| 1 | 336245877 | HM568712.1 | 3 |  | 29 | 336245869 | HM568708.1 | 7 |
| 2 | 336245791 | HM568669.1 | 5 |  | 30 | 336245875 | HM568711.1 | 7 |
| 3 | 336245793 | HM568670.1 | 6 |  | 31 | 336245879 | HM568713.1 | 6 |
| 4 | 336245799 | HM568673.1 | 6 |  | 32 | 336245881 | HM568714.1 | 7 |
| 5 | 336245801 | HM568674.1 | 5 |  | 33 | 336245883 | HM568715.1 | 1 |
| 6 | 336245803 | HM568675.1 | 5 |  | 34 | 336245885 | HM568716.1 | 1 |
| 7 | 336245805 | HM568676.1 | 7 |  | 35 | 336245887 | HM568717.1 | 1 |
| 8 | 336245807 | HM568677.1 | 7 |  | 36 | 336245889 | HM568718.1 | 1 |
| 9 | 336245809 | HM568678.1 | 5 |  | 37 | 336245891 | HM568719.1 | 1 |
| 10 | 336245811 | HM568679.1 | 5 |  | 38 | 336245893 | HM568720.1 | 1 |
| 11 | 336245813 | HM568680.1 | 6 |  | 39 | 336245895 | HM568721.1 | 1 |
| 12 | 336245815 | HM568681.1 | 6 |  | 40 | 336245897 | HM568722.1 | 1 |
| 13 | 336245817 | HM568682.1 | 5 |  | 41 | 336245899 | HM568723.1 | 1 |
| 14 | 336245819 | HM568683.1 | 5 |  | 42 | 336245901 | HM568724.1 | 1 |
| 15 | 336245823 | HM568685.1 | 5 |  | 43 | 336245795 | HM568671.1 | 3 |
| 16 | 336245829 | HM568688.1 | 6 |  | 44 | 336245797 | HM568672.1 | 3 |
| 17 | 336245831 | HM568689.1 | 7 |  | 45 | 336245821 | HM568684.1 | 3 |
| 18 | 336245833 | HM568690.1 | 7 |  | 46 | 336245825 | HM568686.1 | 3 |
| 19 | 336245837 | HM568692.1 | 6 |  | 47 | 336245827 | HM568687.1 | 3 |
| 20 | 336245839 | HM568693.1 | 7 |  | 48 | 336245835 | HM568691.1 | 3 |
| 21 | 336245845 | HM568696.1 | 7 |  | 49 | 336245841 | HM568694.1 | 3 |
| 22 | 336245847 | HM568697.1 | 7 |  | 50 | 336245843 | HM568695.1 | 3 |
| 23 | 336245849 | HM568698.1 | 7 |  | 51 | 336245851 | HM568699.1 | 3 |
| 24 | 336245855 | HM568701.1 | 7 |  | 52 | 336245853 | HM568700.1 | 3 |
| 25 | 336245857 | HM568702.1 | 7 |  | 53 | 336245859 | HM568703.1 | 3 |
| 26 | 336245861 | HM568704.1 | 7 |  | 54 | 336245867 | HM568707.1 | 3 |
| 27 | 336245863 | HM568705.1 | 7 |  | 55 | 336245871 | HM568709.1 | 3 |
| 28 | 336245865 | HM568706.1 | 6 |  | 56 | 336245873 | HM568710.1 | 3 |
