# Supplementary material for: Genetic diversity of the merozoite surface protein-3 gene in Plasmodium falciparum populations in Thailand
Source: Malar J. 2016 Oct 21;15:517. doi: 10.1186/s12936-016-1566-1 (PMC5073822; doi:10.1186/s12936-016-1566-1)
Supplement: Supplementary file 5 — Additional file 5. Maximum-likelihood tree of msp-3 gene from P. falciparum populations in Thailand and Nigeria. [file 12936_2016_1566_MOESM5_ESM.doc]

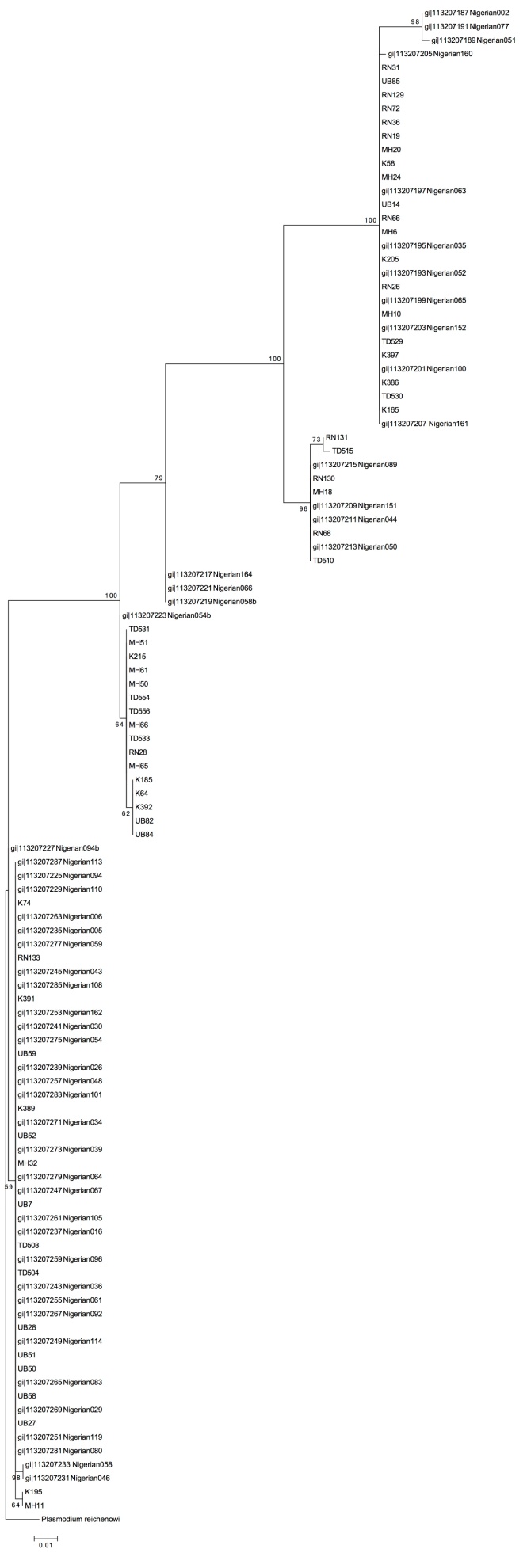


Haplotype 2

Haplotype 8*

Haplotype 1

Haplotype 4 4

Haplotype 3

Haplotype 11*

Haplotype 5

Haplotype 6A and 6B

Haplotype 7

Haplotype 10*

Haplotype 12*

Haplotype 9*

**K1 allele of *msp-3***

**3D7 allele of *msp-3***

**Additional file 5 -** **Maximum-likelihood tree of *msp-3* gene from *P. falciparum* populations in Thailand and Nigeria.** The tree was constructed as described in *Materials and Methods*. The tree was rooted using the sequence of the *P. reichenowi msp-3* gene as an out group [[56](#_ENREF_56)]. Bootstrap values of > 50% are shown. The scale bar indicates nucleotide substitutions per site. Asterisks (*) indicate sequences (Haplotype 8 – Haplotype 12) from Nigeria (n = 50), obtained from Polley *et al.* [[37](#_ENREF_37)]. Haplotype 8 was classified as the 3D7 allele of *msp-3*, while haplotypes 9 – 12 were classified as the K1 allele.
